# The economic burden of angina on households in South Asia

**DOI:** 10.1186/1471-2458-14-179

**Published:** 2014-02-19

**Authors:** Khurshid Alam, Ajay Mahal

**Affiliations:** 1 Monash School of Public Health & Preventive Medicine, Monash University, 99 Commercial Road, The Alfred Centre, Melbourne, Vic, 3004, Australia; 2 Centre for Equity and Health Systems, ICDDR,B, 68 Shaheed Tajuddin Ahmed Sharani, Mohakhali, Dhaka 1212, Bangladesh

**Keywords:** Symptomatic angina, Diagnosed angina, OOP health spending, Catastrophic expenses, Borrowing or selling assets, Employment status, Non-medical consumption expenditure, Households, South Asia

## Abstract

**Background:**

Globally, an estimated 54 million people have angina, 16 million of whom are from the WHO South-East Asia region. Despite the increasing burden of cardiovascular disease (CVD) in South Asia, there is no evidence of an economic burden of angina on households in this region. We investigated the economic burden of angina on households in South Asia.

**Methods:**

We applied a novel propensity score matching approach to assess the economic burden of angina on household out-of-pocket (OOP) health spending, borrowing or selling assets, non-medical consumption expenditure, and employment status of angina-affected individual using nationally representative World Health Survey data from Bangladesh, India, Nepal and Sri Lanka collected during 2002-2003. We used multiple matching methods to match households where the respondent reported symptomatic or diagnosed angina with control households with similar propensity scores.

**Results:**

Angina-affected households had significantly higher OOP health spending per person in the four weeks preceding the survey than matched controls, in Bangladesh (I$1.94, p = 0.04), in Nepal (I$4.68, p = 0.03) and in Sri Lanka (I$1.99, p < 0.01). Nearly half of this difference was accounted for by drug expenditures. Catastrophic spending, defined as the ratio of OOP health spending to total household expenditure in excess of 20%, was significantly higher in angina-affected households relative to matched controls in India (9.60%, p < 0.01), Nepal (4.90%, p = 0.02) and Sri Lanka (9.10%, p < 0.01). Angina-affected households significantly relied on borrowing or selling assets to finance OOP health expenses in Bangladesh (6%, p = 0.03), India (8.20%, p < 0.01) and Sri Lanka (7.80%, p = 0.01). However, impoverishment, non-medical consumption expenditure and employment status of the angina-affected individual remained mostly unaffected. We adjusted our estimates for comorbidities, but limitations on comorbidity data in the WHS mean that our results may be upwardly biased.

**Conclusions:**

Households that had the respondent reporting angina in South Asia face an economic burden of OOP health expenses (primarily on drugs and other outpatient expenses), and tend to rely on borrowing or selling assets. Our analysis underscores the need to protect South Asian households from the financial burden of CVD.

## Background

The World Health Organization (WHO) estimates that 54 million people live with angina pectoris globally, of whom 16 million are from the WHO South-East Asia region. Angina pectoris (or angina) is a chest pain or discomfort that occurs due to lack of oxygen-rich blood in one’s heart muscle [1]. Studies have shown that angina is strongly associated with ischemic heart disease (IHD) that occurs due to obstruction of the coronary arteries [2]. In 2008, nearly 80% of deaths from non-communicable diseases (NCDs) occurred in low-and middle-income countries (LMICs), caused mostly by IHD [3]. Even though angina itself is not a severe health condition like heart attack, people affected by angina will typically need treatment and prevention to avoid further worsening of their health and associated economic consequences. Smokers with angina may need to stop smoking. Regular medication, including beta blockers, angiotensin converting enzyme inhibitors and blood thinning drugs may be required. Because angina often reflects build-up of plaque in blood vessels, it may also necessitate surgical procedures such as angioplasty and cardiac bypass surgery which can be expensive.

The health burden of heart disease in LMICs is acquiring increasing significance [4-6], although the associated economic implications are only recently being appreciated. One study for India showed that out-of-pocket (OOP) payments for hospital care associated with cardiovascular disease (CVD) amounted to almost 30% of annual expenditures among households to which patients belonged [7]. Another study for LMICs showed that OOP expenses for hospital care for CVD exceeded the catastrophic threshold (defined as 40% of non-food expenditure) in 50% of the hospitalized cases in India, China, and Tanzania [8]. However, these analyses focus on hospitalized cases, usually arising from heart attacks. Analyses of the economic consequences of angina on households are relatively scarce in LMICs. Research using World Health Survey (WHS) data for Bangladesh, India, Nepal, Sri Lanka, and Ukraine showed that angina imposed significant OOP health expenditures particularly, for drugs, on households [9,10]. In a developed country context, studies from the United States show that angina exerts major effects on health related quality of life affecting individual’s working ability [11], and chronic angina is associated with significant healthcare costs [12]. In South Asia, where the burden of CVD is likely to rise sharply in future years, an assessment of the economic burden of angina can help in the design of effective policies.

This study seeks to assess the economic burden of angina on households in South Asia, specifically in Bangladesh, India, Nepal and Sri Lanka. The focus on South Asia is driven by the fact that NCDs (particularly heart disease) have attracted much attention as a major source of death and disability in South Asia. In addition, the authors are well informed about the health system contexts in the South Asian region and are in position to make an informed assessment of the ongoing challenges of health financing and delivery in the region.

## Methods

### Data source

The WHS is a set of nationally representative household surveys conducted by the WHO that collected observational data on socioeconomic and demographic characteristics of individuals and households, healthcare financing and healthcare use in 70 low, middle and high income countries during 2002-2003. The WHS data also included disease specific information from one adult member (randomly chosen using Kish Tables) in each household, aged 18 years or over. Sample households were selected based on a random, stratified sampling procedure. The sampling procedure is described in detail elsewhere [13]. The interviews were conducted in person following written consent from the respondent and institutional ethical approval for the survey at each study site. The Monash University Human Research Ethics Committee granted exemption from further ethical review (reference no. CF12/1442-2012000778). Our study included WHS data from four South Asian countries: Bangladesh (5,942 households), India (10,692 households), Nepal (8,822 households) and Sri Lanka (6,805 households), consisting of 32,261 households in total. We could not include WHS data from Pakistan because of the small number of angina cases.

### Propensity score matching approach

Our analysis inquired whether households where a respondent reported angina (angina-affected households) experienced different economic outcomes than households that did not. Because the angina variable can be expected to be non-randomly allocated across respondents, we sought to control for household (and individual respondent) characteristics in undertaking this comparison, using the propensity score matching (PSM) approach [14]. Using PSM, we examined whether OOP health spending, expenditure on drugs and hospitalization, employment and non-medical consumption spending in angina-affected households were different from those of control households. We assessed whether angina-affected households had higher levels of catastrophic OOP spending and medical impoverishment and whether they relied more on borrowing or selling assets for financing OOP health spending than control households.

PSM comprises a two-stage process: in first stage, propensity scores for the treated and control samples are estimated using pre-treatment covariates in a logit model. In the second stage, angina-affected households are matched to control households with similar propensity scores using a matching algorithm. For balance checking, for each covariate used in the regression model that generated the propensity scores, we compared the means between the angina-affected households and matched control households using t-test. We performed analysis using STATA, version 12.1.

### Measurement of treatment variable

We considered three versions of the angina (treatment) variable:

#### Diagnosed angina

Households that had the respondent reporting diagnosed angina were included as ‘treated’ cases and those who did not were taken as ‘control’ cases. Diagnosed angina was defined by affirmative response to the question: “Have you ever been diagnosed with angina or angina pectoris (a heart disease)?” The estimated prevalence of diagnosed angina reported in WHS was 6.58% in Bangladesh, 7.27% in India, 5.45% in Nepal and 2.81% in Sri Lanka.

#### Symptomatic angina

Symptomatic angina was determined on the basis of questions in WHS following the Rose questionnaire [15,16]. Respondents reporting affirmatively to questions about pain and discomfort in the chest when walking; slowing down or stopping when getting pain/discomfort to relieve the pain or taking pain relief medicines; location of the chest pain or discomfort is in the upper middle chest, or lower chest and left arm of the body were determined as having angina. Households that had the respondent reporting these symptoms were included as ‘treated’ cases with the rest as ‘control’ cases. The estimated prevalence of symptomatic angina reported in WHS was 10.34% in Bangladesh, 10.89% in India, 4.39% in Nepal and 3.66% in Sri Lanka.

#### Symptomatic or diagnosed angina

We constructed our third treatment variable where households that had the respondent reporting either symptomatic or diagnosed angina were included as ‘treated’ cases and those who did not were taken as ‘control’ cases. The estimated prevalence of symptomatic or diagnosed angina reported in WHS was 14.98% in Bangladesh, 16.03% in India, 9.31% in Nepal and 6.11% in Sri Lanka. The correlation coefficient for symptomatic and diagnosed angina was 0.17 in Bangladesh, 0.17 in India, 0.06 in Nepal and 0.19 in Sri Lanka.

### Matching variables used to construct propensity scores

#### Individual respondent characteristics

We used age (complete years), indicator of sex (1 if respondent was female, 0 otherwise), indicator of marital status (1 if respondent is currently married or cohabitating, 0 otherwise) and multiple indicators for education of the respondent (1 if primary school completed, 0 otherwise; 1 if secondary school completed, 0 otherwise; 1 if high school completed, 0 otherwise; and 1 if college completed, 0 otherwise). Using information on the most commonly spoken language for each country, we constructed a binary language variable (1 if the respondent spoke that language, 0 otherwise (in Bangladesh Bangla, in India Hindi, in Nepal Nepali and in Sri Lanka Sinhala)). Similarly, using information on majority religious ethnic groups, we constructed a binary indicator of religion (1 if the respondent belonged to the majority religion, 0 otherwise (in Bangladesh Muslim, in India Hindu, in Nepal Hindu, and in Sri Lanka Sinhalese)).

#### Other household members

Socioeconomic and demographic characteristics were used for all household members other than the respondent. These included, specifically, the proportion of females in the household, the proportion of currently married members including those cohabiting in the household, the proportion of children under-five years of age, the proportion of adults (18-59 years), the proportion of elderly (60 years and above) members, and the proportion of members who had completed high school or above. In addition, we used age of the head of household (completed years); indicator of sex of household head (1 if male, 0 otherwise); indicator of marital status of household head (1 if currently married or cohabitating, 0 otherwise); and indicator of education of the household head (1 if completed high school or above, 0 otherwise).

#### Other household characteristics

These included household size, household sample weights, rural-urban indicator (1 if urban, 0 otherwise) and indicators for sampling strata (1 if the household was located within the appropriate stratum, 0 otherwise) used to stratify households regionally for sampling purposes. There were 12 strata in Bangladesh, 12 in India, 71 in Nepal and 33 in Sri Lanka. Multiple indicators for living conditions were also used: improved drinking water sources (1 if the household had access to water from piped sources, protected tube well or bore hole, or protected dug well or protected spring, rainwater, or tanker-truck, 0 otherwise) and improved toilets (1 if the household has toilet facilities with flush or piped sewage system, or flush to septic tank, or poor flush latrine, 0 otherwise) [17]; clean cooking fuel (1 if the household used gas or electricity for cooking, 0 otherwise); improved cooking stove (1 if the household used open fire/stove with chimney or hood, or closed stove with chimney, 0 otherwise); separate space for cooking (1 if the household had separate location as kitchen, 0 otherwise); heating conditions (1 if the household had heating system during cold weather, 0 otherwise); floor type (1 if the floor of the household was hard floor such as tile, cement, brick or wood, 0 otherwise); and wall type (1 if the wall of the household residence was cement, brick, stone or wood, 0 otherwise).

A rudimentary social capital variable (as a proxy for social network) was also constructed based on survey responses to 10 questions in WHS data. First, indicator variables were constructed on respondents’ ability to control important things in life (1 if never or almost never, 0 otherwise); on ability to cope with their responsibilities (1 if never or almost never, 0 otherwise); level of trust in national government (1 if always or most of the time, 0 otherwise); level of trust in local government (1 if always or most of the time, 0 otherwise); feeling safe from crime and violence at home (1 if completely safe or very safe, 0 otherwise); or outside (1 if completely safe or very safe, 0 otherwise); on ability to influence government to address issues of interest (1 if unlimited or a lot of say, 0 otherwise); freedom of expression (1 if completely free or very free, 0 otherwise); voting behaviour in last national election (1 if voted, 0 otherwise); and on being a victim of violent crime (1 if yes, 0 otherwise). Second, a social capital variable was constructed from the 10 indicators based on principal component factor analysis. Social capital positively influences household economic outcomes such as per capita expenditure, assets, savings and access to credit [18].

### Measurement of outcomes

#### OOP health spending

The WHS reported OOP health spending in the four weeks preceding the survey in two ways - one as a single estimate and another in itemized form, with health expenditures separately reported for inpatient care, outpatient care, traditional or alternative sources of care, dentists, medication, healthcare products, laboratory services and others [19]. Single item questions tend to generate significantly lower aggregate expenditures than the sum of multiple disaggregated questions [20,21]. For this reason, and also because of our interest in individual components of OOP spending, we used the sum of item-wise reports of health spending divided by household size (that is, per household member).

#### Spending on drugs

Our measure was OOP spending on drugs per person in the household in the four weeks preceding the survey.

#### Spending on hospitalization

We used household OOP spending on hospitalization expenditure (per member) in the four weeks preceding the survey.

#### Reliance on borrowing or sale of assets to finance health expenditure

The WHS collected information on financing strategies that households used to meet health expenditures in one year preceding the survey [19]. We constructed a binary outcome indicator taking the value 1 if any household reported borrowing from a family or friend or from outside the household, or reporting selling of household items to pay for healthcare, 0 otherwise.

#### Measure of catastrophic OOP health spending

We constructed two measures of catastrophic OOP health spending: an indicator variable that took the value 1 whenever the ratio of household OOP health spending to total expenditure exceeded 20% [22]; and an indicator variable that took the value 1 when the ratio of household OOP health spending to a measure of household’s ‘capacity to pay’ (total expenditure minus subsistence needs) exceeded 40% [23]. A household’s ‘capacity to pay’ (*CTP*) was calculated in the following way:

$$\mathrm{CTP}=\mathrm{TEX}-\mathrm{SE}\left(45-55\right)\;\mathrm{If}\;\mathrm{FEX}>\mathrm{SE}\left(45-55\right)$$

$$\mathrm{CTP}=\mathrm{TEX}-\mathrm{FEX}\;\mathrm{FEX}\;\mathrm{If}\;\mathrm{FEX}<\mathrm{SE}\left(45-55\right)$$

*TEX* denotes total expenditure and *FEX* denotes food expenditure. *SE* stands for subsistence expenditure and is the average food expenditure for households whose food expenditure share of total expenditure is in the 45^th^ to 55^th^ percentile of the total sample of households, adjusted for household size. Household consumption equivalence scales were used rather than the actual household size [23].

#### Impoverishing effect of OOP health spending

Following Wagstaff and van Doorslaer (2003), a household was defined as being impoverished due to illness if its total spending gross of OOP on healthcare exceeded the poverty line, but total spending net of OOP on healthcare was below the poverty line [24]. We used World Bank’s purchasing power parity (PPP) based international poverty line for low-income countries for this purpose, defined as 1.25 international dollars per day per person.

#### Employment

Two indicators for employment status of angina-affected respondent were used. The first was based on whether the respondent was currently working (1 if the respondent was government employee, or non-government employee, or self-employed, or employer, 0 otherwise). The second indicator inquired above the main reason for not working for pay (1 if the respondent was not working due to illness, 0 otherwise).

#### Non-medical consumption expenditure

The WHS data recorded household consumption spending in four weeks preceding the survey in two ways: one as a single aggregate measure and another in itemized form such as food, housing, education, insurance premiums and all other goods [19]. We constructed a measure of non-medical consumption of households by summing up itemized expenditures, excluding medical spending.

All expenditure estimates are reported in international dollar (I$) based on the World Banks’s PPP in 2003.

### Robustness checks

We used multiple matching algorithms – nearest-neighbor, stratification, radius and kernel matching – to compare outcomes for angina-affected households and controls. There is also the possibility that our matching results could be confounded by comorbidities, which can include diabetes, asthma, depression, anemia, arrhythmia (tachycardia), chronic obstructive pulmonary disease, heart failure, hyperthyroidism and renal failure [25-28]. To partially address this, we estimated separate linear regression models (with economic outcomes as the Y variables), an indicator for angina-affected households and an indicator for presence of a comorbid condition (at least one of diabetes, asthma, and depression for which information was available in the WHS dataset) on a dataset consisting only of matched households.

## Results

Table 1 presents summary statistics for the key variables included in the first-stage (propensity score) regression, for (treatment) households that had the respondent reporting symptomatic or diagnosed angina and the subset of matched households (based on nearest-neighbor matching) that had respondents not reporting symptomatic or diagnosed angina. There were no statistically significant differences between treatment households and matched controls (based on nearest-neighbor matching) on age, sex, marital status and education level of the angina-affected individual, other household members, household head and basic household characteristics within any of the four South Asian countries. Although not presented in Table 1, separate analyses for matching involving diagnosed angina (only) as treatment, and symptomatic angina (only) as treatment did not indicate statistically different values between the pre-treatment covariates for treatment and control households.

**Table 1:** Summary of key matching variables by symptomatic or diagnosed angina-affected and control households in South Asia, 2002-2003

**Matching variables**	**Bangladesh**		**India**		**Nepal**		**Sri Lanka**	
	**Treated**	**Controls-matched**	**Treated**	**Controls-matched**	**Treated**	**Controls-matched**	**Treated**	**Controls-matched**
***Angina-affected individual***								
Age (mean)	42.30 (41.17, 43.43)	42.31 (41.03, 43.59)	43.03 (42.11, 43.95)	43.37 (42.34, 44.40)	45.19 (43.94, 46.44)	44.50 (43.16, 45.84)	44.96 (43.32, 46.60)	45.73 (43.84, 47.62)
Sex: female (%)	61.28 (57.76, 64.80)	62.91 (59.12, 66.70)	57.07 (54.17, 59.97)	56.35 (53.06, 59.64)	57.96 (54.00, 61.92)	58.12 (53.97, 62.27)	54.37 (48.91, 59.83)	52.19 (46.21, 58.17)
Education level: high school and above (%)	3.40 (2.09, 4.71)	3.12 (1.76, 4.48)	5.28 (3.97, 6.59)	5.99 (4.41, 7.57)	3.01 (1.64, 4.38)	3.01 (1.57, 4.45)	6.25 (3.60, 8.90)	6.87 (3.84, 9.90)
Marital status: currently married (%)	78.40 (75.43, 81.37)	80.03 (76.89, 83.17)	80.59 (78.27, 82.91)	80.23 (77.59, 82.87)	87.44 (84.78, 90.10)	88.78 (86.13, 91.43)	75.94 (71.26, 80.62)	78.12 (73.17, 83.07)
***Other non-angina household members***								
Children under-five years of age (%)	13.08 (10.64,15.52)	12.68 (10.07, 15.29)	9.10 (7.41, 10.79)	9.28 (7.35, 11.21)	11.92 (9.32, 14.52)	12.54 (9.75, 15.33)	8.01 (5.04, 10.98)	8.71 (5.33, 12.09)
Adult members (%)	39.89 (36.35, 43.43)	39.34 (35.51, 43.17)	40.20 (37.33, 43.07)	40.22 (36.97, 43.47)	37.77 (33.88, 41.66)	36.62 (32.57, 40.67)	48.54 (43.06, 54.02)	48.41 (42.43, 54.39)
Elderly members (%)	7.93 (5.98, 9.88)	8.11 (5.97, 10.25)	18.28 (16.01, 20.55)	17.29 (14.78, 19.80)	8.58 (6.33, 10.83)	8.71 (6.34, 11.08)	9.18 (6.02, 12.34)	9.52 (6.01, 13.03)
Sex: female (%)	46.96 (43.35, 50.57)	47.80 (43.88, 51.72)	52.59 (49.66, 55.52)	52.40 (49.09, 55.71)	50.76 (46.75, 54.77)	50.47 (46.26, 54.68)	48.21 (42.74, 53.68)	48.56 (42.58, 54.54)
Education level: high school and above (%)	6.93 (5.10, 8.76)	6.66 (4.70, 8.62)	14.85 (12.77, 16.93)	14.76 (12.41, 17.11)	5.69 (3.83, 7.55)	5.53 (3.61, 7.45)	18.75 (14.47, 23.03)	20.94 (16.07, 25.81)
Marital status: currently married (%)	36.71 (33.23, 40.19)	36.97 (33.18, 40.76)	37.10 (34.27, 39.93)	37.10 (33.89, 40.31)	37.99 (34.10, 41.88)	37.42 (33.35, 41.49)	34.87 (29.65, 40.09)	36.13 (30.38, 41.88)
***Characteristics of household head***								
Age (mean)	43.31 (42.24, 44.38)	42.69 (41.58, 43.80)	45.52 (44.69, 46.35)	45.58 (44.61, 46.55)	46.10 (44.94, 47.26)	45.97 (44.70, 47.24)	48.89 (47.32, 50.46)	49.68 (47.86, 51.5)
Sex: male-headed household (%)	88.72 (86.43, 91.01)	86.68 (84.01, 89.35)	91.23 (89.57, 92.89)	92.04 (90.24, 93.84)	83.42 (80.44, 86.4)	84.25 (81.19, 87.31)	80.62 (76.29, 84.95)	80.62 (75.89, 85.35)
Education level: high school and above (%)	5.43 (3.79, 7.07)	5.03 (3.32, 6.74)	6.26 (4.84, 7.68)	5.99 (4.41, 7.57)	3.35 (1.91, 4.79)	3.01 (1.57, 4.45)	4.37 (2.13, 6.61)	5.31 (2.63, 7.99)
Marital status: currently married (%)	85.60 (83.06, 88.14)	85.73 (82.99, 88.47)	85.96 (83.92, 88.00)	86.94 (84.70, 89.18)	90.28 (87.90, 92.66)	91.12 (88.73, 93.51)	80.94 (76.64, 85.24)	82.50 (77.95, 87.05)
***Characteristics of household***								
Household size (mean)	5.56 (5.40, 5.72)	5.40 (5.23, 5.57)	6.27 (6.12, 6.42)	6.32 (6.16, 6.48)	5.37 (5.18, 5.56)	5.37 (5.20, 5.54)	4.50 (4.30, 4.70)	4.51 (4.31, 4.71)
Location: urban (%)	21.19 (18.24, 24.14)	19.56 (16.45, 22.67)	14.40 (12.34, 16.46)	14.67 (12.32, 17.02)	10.89 (8.39, 13.39)	10.55 (7.97, 13.13)	14.37 (10.53, 18.21)	13.44 (9.36, 17.52)
Improved water source (%)	96.19 (94.81, 97.57)	96.19 (94.69, 97.69)	83.81 (81.65, 85.97)	83.54 (81.08, 86.00)	90.62 (88.28, 92.96)	92.63 (90.43, 94.83)	76.87 (72.25, 81.49)	74.06 (68.81, 79.31)
Improved latrine (%)	16.44 (13.76, 19.12)	15.35 (12.52, 18.18)	16.90 (14.70, 19.10)	17.44 (14.92, 19.96)	23.62 (20.21, 27.03)	21.94 (18.46, 25.42)	61.87 (56.55, 67.19)	61.87 (56.05, 67.69)
Clean cooking fuel (%)	0.81 (0.16, 1.46)	0.68 (0.04, 1.32)	2.59 (1.66, 3.52)	3.13 (1.97, 4.29)	1.67 (0.64, 2.70)	1.67 (0.59, 2.75)	0.31 (-0.30, 0.92)	0.00 (0.00, 0.00)
Improved cooking stove (%)	1.90 (0.91, 2.89)	2.72 (1.44, 4.00)	16.90 (14.70, 19.10)	19.14 (16.53, 21.75)	6.87 (4.84, 8.90)	8.37 (6.04, 10.70)	30.00 (24.98, 35.02)	34.69 (28.99, 40.39)
**Sample**	**736**	**624**	**1118**	**872**	**597**	**543**	**320**	**268**

*Notes*: Un-weighted estimates are based on authors’ calculations using raw data from the World Health Survey conducted during 2002-2003. The data presented refer to the households, which responded to the survey question on whether or not a household member experienced any angina which includes diagnosed or symptomatic angina. 95% confidence intervals are reported in parentheses underneath each mean/proportion. Statistically significant difference between the treatment and matched controls households are shown at the significance level of 5%* based on the nearest-neighbor algorithm.

We did, however, find statistically significant differences in economic outcomes between households that had a respondent reporting symptomatic or diagnosed angina and their matched controls. Table 2 reports these findings for the nearest-neighbor matching method. Per person OOP health spending was generally higher in households that had the respondent reporting symptomatic or diagnosed angina relative to matched controls across all four South Asian countries we studied, and this difference was statistically significant in Bangladesh (I$8.80 versus I$6.86, p = 0.04), Nepal (I$9.65 versus I$4.97, p = 0.03) and Sri Lanka (I$4.22 versus I$2.23, p < 0.01). Nearly half of this difference was accounted for by drug expenditures which were significantly higher in Nepal (I$6.71 versus I$3.27, p = 0.01) and Sri Lanka (I$1.94 versus I$1.00, p < 0.01), relative to the matched control group. Per person OOP hospitalization expenditure was significantly (p = 0.05) different between the treated and control groups only in Sri Lanka. Our data also indicate that the proportion of households reporting borrowing or sale of assets to finance their OOP health spending was between 4%-8% higher in treatment households relative to matched controls in all four countries, and only in Nepal were the differences not statistically significant at 5% level. In Sri Lanka, the proportion of treatment households reporting borrowing or sale of assets was, at 21%, the lowest among the four countries studied.

**Table 2:** Differences in economic outcomes between symptomatic or diagnosed angina-affected households and control households in South Asia, 2002-2003

**Economic outcome indicators**	**Bangladesh**			**India**			**Nepal**			**Sri Lanka**		
	**Treated**	**Controls-matched**	***p*-value**	**Treated**	**Controls-matched**	***p*-value**	**Treated**	**Controls-matched**	***p*-value**	**Treated**	**Controls-matched**	***p*-value**
Per person OOP health spending in last four weeks (I$)	8.80 (7.39, 10.21)	6.86 (5.76, 7.96)	0.04*	8.25 (6.94, 9.56)	6.80 (5.00, 8.60)	0.25	9.65 (5.79, 13.51)	4.97 (3.59, 6.35)	0.03*	4.22 (3.16, 5.28)	2.23 (1.42, 3.04)	<0.01*
Per person expenditure on medicine in last four weeks (I$)	5.40 (4.77, 6.03)	4.60 (3.80, 5.40)	0.16	4.31 (3.30, 5.32)	3.45 (2.40, 4.50)	0.30	6.71 (4.16, 9.26)	3.27 (2.57, 3.97)	0.01*	1.94 (1.46, 2.42)	1.00 (0.68, 1.32)	<0.01*
Per person hospitalization expenses in last four weeks (I$)	0.96 (-0.01, 1.93)	0.24 (0.04, 0.44)	0.16	1.44 (0.87, 2.01)	1.66 (0.35, 2.97)	0.80	1.16 (0.15, 2.17)	0.46 (-0.01, 0.93)	0.22	0.92 (0.22, 1.62)	0.18 (-0.04, 0.40)	0.05*
Borrowing or selling assets to finance health expenditure in last one year (%)	46.06 (42.46, 49.66)	40.08 (36.23, 43.93)	0.03*	51.79 (48.86, 54.72)	43.56 (40.27, 46.85)	<0.01*	57.62 (53.66, 61.58)	53.60 (49.41, 57.79)	0.18	21.25 (16.77, 25.73)	13.44 (9.36, 17.52)	0.01*
OOP health spending share of total household expenditure at 20% cut-off	28.40 (25.14, 31.66)	24.59 (21.21, 27.97)	0.13	28.26 (25.62, 30.9)	18.69 (16.10, 21.28)	<0.01*	18.26 (15.16, 21.36)	13.40 (10.53, 16.27)	0.02*	13.44 (9.70, 17.18)	4.37 (1.92, 6.82)	<0.01*
OOP health spending share of household’s ‘capacity to pay’ at 40% cut-off	39.40 (35.87, 42.93)	35.87 (32.11, 39.63)	0.20	33.00 (30.24, 35.76)	26.30 (23.38, 29.22)	<0.01*	21.27 (17.99, 24.55)	16.75 (13.61, 19.89)	0.05*	21.87 (17.34, 26.40)	11.87 (8.00, 15.74)	<0.01*
Ratio of OOP health spending and total household expenditure (%)	15.73 (13.10, 18.36)	13.75 (11.05, 16.45)	0.02*	15.10 (13.00, 17.20)	11.52 (9.40, 13.64)	<0.01*	9.86 (7.47, 12.25)	7.61 (5.38, 9.84)	0.01*	7.91 (4.95, 10.87)	4.49 (2.01, 6.97)	<0.01*
Ratio of OOP medicine expenses and total household expenditure (%)	11.28 (8.99, 13.57)	10.07 (7.71, 12.43)	0.05*	8.62 (6.97, 10.27)	6.89 (5.21, 8.57)	<0.01*	7.97 (5.80, 10.14)	5.84 (3.87, 7.81)	<0.01*	3.96 (1.82, 6.10)	2.19 (0.44, 3.94)	<0.01*
Impoverishment due to OOP health payments (%)	12.63 (10.23, 15.03)	11.82 (9.29, 14.35)	0.66	10.20 (8.43, 11.97)	8.32 (6.49, 10.15)	0.18	8.37 (6.15, 10.59)	6.20 (4.17, 8.23)	0.16	5.31 (2.85, 7.77)	1.87 (0.25, 3.49)	0.03*
Employment status of angina-affected individual (%)	40.76 (37.21, 44.31)	38.45 (34.63, 42.27)	0.41	55.10 (52.18, 58.02)	56.08 (52.79, 59.37)	0.68	67.50 (63.74, 71.26)	70.52 (66.68, 74.36)	0.28	39.69 (34.33, 45.05)	40.62 (34.74, 46.5)	0.83
Unemployed due to illness (%)	2.72 (1.54, 3.90)	0.27 (-0.14, 0.68)	<0.01*	5.81 (4.44, 7.18)	4.29 (2.95, 5.63)	0.14	4.86 (3.14, 6.58)	2.85 (1.45, 4.25)	0.07	8.12 (5.13, 11.11)	3.44 (1.26, 5.62)	0.02*
Per person non-medical consumption expenditure in last four weeks (I$)	46.78 (36.51, 57.05)	39.81 (33.24, 46.38)	0.25	34.02 (30.95, 37.09)	37.30 (29.12, 45.48)	0.42	47.65 (43.69, 51.61)	42.41 (39.60, 45.22)	0.04*	37.72 (35.46, 39.98)	40.60 (37.43, 43.77)	0.18
**Sample**	**736**	**624**	**-**	**1118**	**872**	**-**	**597**	**543**	**-**	**320**	**268**	**-**

*Notes*: Un-weighted estimates are based on authors’ calculations using raw data from the World Health Survey conducted during 2002-2003 and using nearest-neighbor matching. The data presented refer to the households, which responded to the survey question on whether or not a household member experienced any angina which includes diagnosed or symptomatic angina. 95% confidence intervals are reported in parentheses underneath each mean/proportion. Considering the sample used in the nearest-neighbor matching statistically significant difference between the treatment and matched controls are shown at the significance level of 5%*. All expenditure estimates are in international dollar based on the World Banks’s purchasing power parity in 2003. For impoverishing effect the World Bank’s international poverty line for low income countries (1.25 international dollar per person per day) is used.

The share of households reporting catastrophic health spending was higher in treatment households compared to matched controls by 3%-10% in all countries, but statistical significance was not always achieved. When catastrophic spending was defined as OOP health spending exceeding 20% of total household expenditure, it was significantly higher in angina-affected households (relative to matched controls) in India (28.26% versus 18.69%, p < 0.01), Nepal (18.26% versus 13.40%, p = 0.02) and Sri Lanka (13.44% versus 4.37%, p < 0.01); although lower in absolute magnitude, the results were in the same direction when catastrophic spending was instead defined as the ratio of OOP health spending to household’s ‘capacity to pay’ in excess of 40%, also in the same set of countries. We also used the ratio OOP health spending to total household expenditure and the ratio of OOP drug expenditures to total household expenditure as outcome measures. Here again, the differences between angina-affected households and matched control households were significantly higher, ranging from 2%-4% for the OOP spending to total household expenditure, and 1%-2% for the ratio of OOP spending on drugs to total household expenditure.

Angina-affected households also reported higher levels of impoverishment due to OOP spending relative to matched controls (with the difference ranging from 0.80% to 3.50% of households), although the difference was statistically significant (p = 0.03) only in Sri Lanka. Employment of the angina-affected individual was generally lower compared to their matched counterparts in most South Asian countries; however, none of the differences were statistically significant at the 5% level. Because there could be a number of reasons for why people are working (or not), particularly women who have lower workforce participation rates, we also considered an alternative outcome measure: whether a person reported not working due to illness. Generally, the share of respondents not working due to illness were higher among those reporting angina, relative to matched counterparts in all four countries, by 1.50% to 4.70% and these differences were statistically significant (at 5% level) in Bangladesh and Sri Lanka; and Nepal at the 10% level of significance. Differences in per person non-medical consumption expenditure between angina-affected and control households were statistically indistinguishable from zero in Bangladesh, India and Sri Lanka. In Nepal, however, non-medical consumption expenditure was actually higher in angina-affected households relative to matched controls (I$47.65 versus I$42.41, p = 0.04).

Table 3 reports our results for three alternative indicators of angina-affected (treatment) households –diagnosed with angina, having symptoms of angina, and having either of the two. The set of treatment and matched control households differs for the three indicators so the estimates of the economic burden are not directly comparable across the different angina definitions. Nonetheless, there are some similarities in outcomes across the different angina indicators. OOP spending overall and OOP spending on drugs are higher in treatment households, relative to matched controls, even if not always statistically significant. The differences between angina-affected households and matched controls are sharper in many cases (that is, become statistically different) when overall OOP healthcare spending and OOP spending on drugs are taken as a share of aggregate household spending. Differences in catastrophic OOP spending are generally significantly higher in angina-affected households relative to matched controls, with the exception of Bangladesh where no differences were observed, similar to results in Table 2; and with the exception of Sri Lanka, no differences in medical impoverishment were observed between angina-affected and matched control households.

**Table 3:** Differences in economic outcomes between angina-affected households and control households in South Asia, 2002-2003: Results for different definitions of angina-affected household

**Economic outcome indicators**	**Bangladesh**			**India**			**Nepal**			**Sri Lanka**		
	**Angina (any)**	**Angina (symptom)**	**Angina (diagnosed)**	**Angina (any)**	**Angina (symptom)**	**Angina (diagnosed)**	**Angina (any)**	**Angina (symptom)**	**Angina (diagnosed)**	**Angina (any)**	**Angina (symptom)**	**Angina (diagnosed)**
Per person OOP health spending in last four weeks (I$)	1.94* (0.04)	1.50 (0.15)	0.49 (0.75)	1.45 (0.25)	1.25 (0.32)	4.91* (<0.01)	4.68* (0.03)	4.14 (0.12)	2.61 (0.65)	1.99* (<0.01)	4.47 (0.18)	5.23 (0.15)
Per person expenditure on medicine in last four weeks (I$)	0.80 (0.16)	0.66 (0.23)	0.70 (0.23)	0.87 (0.30)	0.77 (0.36)	1.99 (0.08)	3.45* (0.01)	3.59 (0.12)	0.52 (0.89)	0.94* (<0.01)	0.22 (0.61)	0.92* (0.04)
Per person hospitalization expenses in last four weeks (I$)	0.72 (0.16)	0.56 (0.37)	-0.45 (0.71)	-0.21 (0.80)	-0.20 (0.79)	1.51* (0.01)	0.70 (0.22)	0.53 (0.28)	0.16 (0.92)	0.74* (0.05)	3.66 (0.27)	4.46 (0.22)
Borrowing or selling assets to finance health expenditure in last one year (%)	6.00* (0.03)	2.20 (0.53)	0.90 (0.82)	8.20* (<0.01)	6.40* (0.02)	9.70* (<0.01)	4.00 (0.18)	8.80* (0.04)	1.80 (0.65)	7.80* (0.01)	10.40* (0.01)	7.70 (0.10)
OOP health spending share of total household expenditure at 20% cut-off (%)	3.80 (0.13)	<0.01 (1.00)	1.80 (0.63)	9.60* (<0.01)	2.50 (0.28)	13.30* (<0.01)	4.90* (0.02)	7.50* (0.01)	5.80* (0.04)	9.10* (<0.01)	3.30 (0.31)	9.50* (<0.01)
OOP health spending share of household’s ‘capacity to pay’ at 40% cut-off (%)	3.50 (0.20)	2.00 (0.56)	-1.80 (0.65)	6.70* (<0.01)	0.60 (0.80)	10.10* (<0.01)	4.50* (0.05)	8.40* (<0.01)	7.00* (0.02)	10.00* (<0.01)	8.20* (0.05)	8.90* (0.04)
Ratio of OOP health spending and total household expenditure (%)	2.00* (0.02)	0.40 (0.70)	1.10 (0.32)	3.60* (<0.01)	1.40 (0.15)	5.40* (<0.01)	2.20* (0.01)	4.00* (<0.01)	3.70* (<0.01)	3.40* (<0.01)	2.10 (0.08)	3.30* (<0.01)
Ratio of OOP medicine expenses and total household expenditure (%)	1.20* (0.05)	0.40 (0.59)	0.40 (0.60)	1.70* (<0.01)	0.40 (0.61)	2.00* (0.02)	2.10* (<0.01)	3.70* (<0.01)	2.70* (<0.01)	1.80* (<0.01)	0.30 (0.68)	2.00* (<0.01)
Impoverishment due to OOP health payments (%)	0.80 (0.66)	-3.10 (0.18)	1.50 (0.60)	1.90 (0.18)	0.10 (0.94)	0.20 (0.92)	2.20 (0.16)	2.60 (0.25)	2.40 (0.19)	3.40* (0.03)	2.20 (0.27)	4.70* (0.02)
Employment status of angina-affected individual (%)	2.30 (0.41)	1.50 (0.65)	-1.50 (0.71)	-1.00 (0.68)	3.50 (0.19)	-3.00 (0.37)	-3.00 (0.28)	1.90 (0.62)	-1.50 (0.68)	-0.90 (0.83)	-5.50 (0.30)	-1.80 (0.76)
Unemployed due to illness (%)	2.40* (<0.01)	1.50 (0.08)	2.90* (0.01)	1.50 (0.14)	0.50 (0.65)	3.20* (0.02)	2.00 (0.07)	2.60 (0.11)	1.50 (0.29)	4.70* (0.02)	7.10* (<0.01)	5.30 (0.06)
Per person non-medical consumption expenditure in last four weeks (I$)	6.97 (0.25)	-0.17 (0.97)	19.87 (0.07)	-3.27 (0.42)	3.09 (0.09)	5.37 (0.07)	5.24* (0.04)	-9.66 (0.28)	7.45* (0.04)	-2.88 (0.18)	-2.10 (0.49)	-2.23 (0.39)
**Treatment (control)**	**736 (624)**	**458 (414)**	**339 (308)**	**1118 (872)**	**793 (677)**	**503 (453)**	**597 (543)**	**308 (283)**	**328 (310)**	**320 (268)**	**183 (168)**	**169 (148)**

*Notes*: Coefficients are the average treatment effects derived from the nearest-neighbor matching. For each coefficient statistical significant difference between the treatment and matched controls is shown at the level of 5%*. Each p-value is reported in parenthesis below each estimate of the difference in outcomes between angina-affected and matched control households. All expenditure estimates are in international dollar based on the World Banks’s purchasing power parity in 2003. For impoverishing effect the World Bank’s international poverty line for low income countries (1.25 international dollar per person per day) is used.

There were no differences in employment status of angina-affected respondents between treatments and matched control households in any country, irrespective of the angina indicators used. However, unemployment due to ill health was higher among respondents by about 1%-7%, with statistically significant differences between treatment and matched control households in Sri Lanka and Bangladesh.

Finally, household reliance on borrowing or sale of assets to finance OOP healthcare spending was greater in angina-affected households – by between 1% and 10% - relative to matched controls, but the differences were not always statistically significant. The largest differences in borrowing or sale of assets among angina-affected and control households were observed in India and Sri Lanka, ranging between 6% and 10%. No discernible pattern was visible in the estimates the outcomes across the three different indicators of angina in Table 3.

Table 4 reports results based on regressions on matched data using the nearest-neighbor matching, with our outcomes variables as the Y variables, and as regressors, an indicator for angina and an indicator for whether the respondent had comorbidity associated with CVD (diabetes or asthma or depression). The results in Table 4 show that although many of the differences with matched controls become lower and become statistically insignificant in some cases, the direction of results and our main conclusions are unchanged.

**Table 4:** Differences in economic outcomes between angina-affected and control households in South Asia, 2002-2003: Implications of controlling for comorbid conditions

**Economic outcome indicators**	**Bangladesh**			**India**			**Nepal**			**Sri Lanka**		
	**Angina (any)**	**Angina (symptom)**	**Angina (diagnosed)**	**Angina (any)**	**Angina (symptom)**	**Angina (diagnosed)**	**Angina (any)**	**Angina (symptom)**	**Angina (diagnosed)**	**Angina (any)**	**Angina (symptom)**	**Angina (diagnosed)**
Per person OOP health spending in last four weeks (I$)	1.55 (0.11)	1.46 (0.16)	0.18 (0.91)	0.73 (0.54)	0.39 (0.75)	4.42* (<0.01)	4.18 (0.06)	4.26 (0.14)	2.44 (0.67)	1.84* (0.01)	2.93 (0.42)	4.33 (0.27)
Per person expenditure on medicine in last four weeks (I$)	0.38 (0.48)	0.64 (0.24)	0.42 (0.47)	0.35 (0.66)	0.22 (0.80)	1.73 (0.16)	3.19* (0.03)	3.34 (0.19)	0.56 (0.88)	0.86* (<0.01)	0.05 ( 0.90)	0.89* (0.05)
Per person hospitalization expenses in last four weeks (I$)	0.80 (0.15)	0.61 (0.35)	-0.33 (0.78)	-0.05 (0.95)	-0.45 (0.52)	1.55* (0.02)	0.68 (0.26)	0.84 (0.13)	0.20 (0.91)	0.77 (0.07)	2.28 (0.53)	3.71 (0.34)
Borrowing or selling assets to finance health expenditure in last one year (%)	3.45 (0.21)	1.92 (0.57)	-0.77 (0.85)	4.94* (0.03)	3.30 (0.21)	3.87 (0.25)	3.05 (0.31)	9.20* (0.03)	0.40 (0.92)	8.58* (<0.01)	10.51* (0.01)	6.75 (0.14)
OOP health spending share of total household expenditure at 20% cut-off (%)	2.87 (0.24)	-1.44 (0.63)	-0.27 (0.94)	6.34* (<0.01)	2.29 (0.32)	11.26* (<0.01)	5.36* (0.01)	8.37* (<0.01)	4.83 (0.09)	8.50* (<0.01)	4.98 (0.14)	8.58* (<0.01)
OOP health spending share of household’s ‘capacity to pay’ at 40% cut-off (%)	2.94 (0.28)	0.49 (0.88)	-4.78 (0.22)	3.51 (0.11)	-0.24 (0.92)	8.20* (<0.01)	5.08* (0.03)	9.59* (<0.01)	6.51* (0.03)	11.09* (<0.01)	9.85* (0.02)	8.46* (0.05)
Ratio of OOP health spending and total household expenditure (%)	1.47 (0.07)	0.18 (0.85)	0.43 (0.70)	2.27* (<0.01)	0.91 (0.34)	4.41* (<0.01)	2.22* (0.01)	3.75* (<0.01)	3.32* (<0.01)	3.27* (<0.01)	2.29 (0.06)	2.99* (0.01)
Ratio of OOP medicine expenses and total household expenditure (%)	0.76 (0.22)	0.29 (0.70)	0.04 (0.96)	1.00 (0.11)	-0.03 (0.97)	1.77 (0.06)	2.12* (<0.01)	3.45* (0.01)	2.34* (0.01)	1.63* (<0.01)	0.07 (0.92)	1.93* (0.01)
Impoverishment due to OOP health payments (%)	1.13 (0.53)	-3.47 (0.12)	1.83 (0.52)	0.93 (0.50)	0.46 (0.77)	-0.78 (0.72)	1.95 (0.22)	1.89 (0.42)	2.04 (0.28)	3.01 (0.07)	2.32 (0.26)	4.77* (0.03)
Employment status of angina-affected individual (%)	2.06 (0.45)	0.52 (0.88)	0.78 (0.85)	-1.29 (0.58)	2.82 (0.29)	-0.83 (0.80)	-1.00 (0.72)	2.51 (0.54)	0.54 (0.88)	0.62 (0.88)	-4.04 (0.45)	1.03 (0.85)
Unemployed due to illness (%)	1.93* (<0.01)	1.13 (0.20)	2.21 (0.08)	0.40 (0.70)	-0.06 (0.95)	2.70 (0.07)	2.03 (0.08)	1.70 (0.32)	1.14 (0.44)	3.31 (0.10)	3.91 (0.15)	4.34 (0.13)
Per person non-medical consumption expenditure in last four weeks (I$)	7.60 (0.24)	1.47 (0.72)	19.27 (0.10)	-2.76 (0.45)	1.65 (0.38)	2.77 (0.39)	5.59* (0.03)	-15.27 (0.08)	7.47* (0.04)	-4.67 (0.02)*	-2.84 (0.35)	-2.82 (0.26)
**Sample**	**1360**	**872**	**647**	**1990**	**1470**	**956**	**1140**	**591**	**638**	**588**	**351**	**317**

*Notes*: Regression coefficients are derived using data sets consisting of only matched households using the nearest-neighbor method and coefficients are controlled for co-morbid conditions such as diabetes, asthma and depression. Each p-value is in parenthesis. For each coefficient statistical significance is shown at the level of 5%*. All expenditure estimates are in international dollar based on the World Banks’s purchasing power parity in 2003. For impoverishing effect the World Bank’s international poverty line for low income countries (1.25 international dollar per person per day) is used.

## Discussion

Our analysis suggests that angina-affected households in South Asia face a higher economic burden than similar control households, based on propensity score comparisons. One aspect of this burden is OOP spending on health services, primarily outpatient care and medicines. Our analysis points to drug expenditures as a major source of financial worry for the South Asian households that have members with angina. Although OOP hospitalization expenditures were not overall a significant concern when considering the economic consequences of angina in Bangladesh, India and Nepal; Sri Lankan angina-affected households were an exception and this may indicate greater awareness of ill-health and reliance on healthcare use in Sri Lanka relative to its South Asian neighbors [29,30].

Our analysis also points to a greater reliance on borrowing or sale of assets to finance OOP healthcare by angina-affected households relative to controls. This is of concern since reliance on borrowing or sale of assets can have long-term implications for the economic future of household members if, for instance, there are high costs of borrowing or if income-earning assets are sold. The findings on borrowing or sale of assets may reflect the limited nature of formal health insurance cover (or good quality subsidized public services) available to populations in South Asia [31]. Bangladesh and Nepal face well known problems in access to public services or other forms of insurance. The recent expansion of publicly financed insurance cover in India has focused on inpatient care [32], so angina-related OOP expenses will continue to a source of concern to households in that country. However, the relatively large effect of angina on borrowing or sale of assets among households in Sri Lanka affected by angina was a surprise, however, given its highly subsidized public sector services. One explanation for this finding is that much of the added expenditure for angina is for drugs for which Sri Lankans are still likely to pay OOP payments [33]. Another is the increasing role of private sector healthcare providers in Sri Lanka, who are usually paid for OOP health payments given the limited private insurance coverage for outpatient services [34]. Nonetheless, it is noteworthy that Sri Lankan households that have a member reporting diagnosed or symptom-based angina have the lowest rates of borrowing or asset sales among the four countries in the region (21%, compared to Bangladesh, India and Nepal, where the rates range from 46% to 58%).

Previous studies have suggested that illness is associated with reduced labor supply and earnings [35-37]. Our results indicate that these effects are small for respondents with angina - ranging from 1%-5% in Bangladesh, India, Nepal and Sri Lanka. We suspect these labor supply effects may be small, possibly because the angina-affected individuals can continue their usual work activities either because they need to, or because they are simply unaware of their health status. In Sri Lanka, unemployment due to illness is relatively larger, of the order of 5%, relative to matched controls and this again, may reflect higher levels of health awareness and health seeking behavior in that country’s population. Indeed, when we used diagnosed angina as the indicator, we also found that unemployment due to illness increased among respondents in Bangladesh, India, Nepal and Sri Lanka, relative to matched controls.

Illness is likely to be associated with changes in non-medical consumption of low-income households, although not all analyses reach this conclusion [38-42]. We did not find any differences in non-medical consumption expenditure between angina-affected households and controls in Bangladesh, India and Sri Lanka. In Nepal, on the other hand, there was a statistically significant difference but in this case, non-medical consumption expenditure was higher among angina-affected households. It is difficult to interpret these findings since some authors suggest that food consumption will be higher in households with sick individuals owing to the need for special diets [43,44]. In the WHS data we used, one further source of confounding is the explicit exclusion of transportation expenses for accessing health services from reported OOP expenses in the WHS questionnaire. If, as we expect, transportation expenses for healthcare ended up in non-medical expenditures that could be another reason for higher-reported non-medical expenses among angina-affected households in Nepal. However, WHS data are insufficiently detailed to permit a fuller assessment of these hypotheses.

Our study has obvious limitations. Our analysis was based on self-reported household expenditure data which is subject to measurement error [45,46]. Respondents are tend to provide socially acceptable answers when they are asked frequencies or amounts, they may rely on best estimates rather than recalling and counting [47]. The results of a test-retest study of the WHS specifically found that respondents in this survey tended to under-report total household expenditure, and over-report OOP health expenditure [21].

Our treatment variables were self-reported symptom-based angina and self-reported diagnosed angina, which are likely to be functions of levels of health awareness and health seeking behaviour. This might lead to multiple biases in our results. For example, some unreported angina cases end up in control households. If so, our estimates of economic burden would provide a lower bound of the economic implications of angina for households in South Asia. On the other hand, awareness and diagnosis might be associated with more severe cases of CVD or greater propensity for health services use. We tried to control for this possibility by taking account of comorbidities for which information was available in the WHS data, but only limited information on such comorbidities were available. In this case, our analysis could be overestimating the economic burden of the average angina case in South Asia. More generally, unobservable factors that lead might to exclusion of a pool of households at risk for angina from matched controls could bias our estimated economic burden of angina upwards if the excluded households are also at risk for acquiring other serious illnesses and increased health spending.

## Conclusions

Ours is the first paper to explore the economic burden of angina on households in South Asia. We found that the burden of OOP spending could be significant on households that have a member with angina, and that this burden arises primarily due to outpatient care, including spending on medicines. Our analysis also suggests that angina may increase the risk of not working due to illness, although this effect appears to be small, with the exception of Sri Lanka. However, we did not find any adverse effects on non-medical consumption expenditure. Households where a respondent reported angina also tended to report more reliance on borrowing or sale of assets to finance OOP spending relative to controls. These findings underline the need for policymakers to consider measures to protect households against not just the hospital expenses associated with NCDs, but also in outpatient care settings, where conditions such as angina are more likely to be managed.

## Competing interests

The authors declare that they have no competing interests.

## Authors’ contributions

KA and AM conceptualized the design of the study. KA has analyzed data and drafted initial report receiving guidance from AM. Both the authors contributed to writing the final version of the paper.

## Authors’ information

KA is a doctoral student, and AM is KA’s principal adviser and the Alan & Elizabeth Finkel Chair in Global Health.

## Pre-publication history

The pre-publication history for this paper can be accessed here:

http://www.biomedcentral.com/1471-2458/14/179/prepub
